# Evaluation and Comparison for the Efficacy of 810 nm Diode Laser, Nano Carbonate Apatite and Their Combination Over Dentinal Tubules Occlusion: An In Vitro Scanning Electron Microscopic Study

**DOI:** 10.7759/cureus.55718

**Published:** 2024-03-07

**Authors:** Kunal Keshaw, Anita Raikar, Pushpa SP

**Affiliations:** 1 Periodontology, Bharati Vidyapeeth Deemed to Be University Dental College and Hospital, Sangli, IND; 2 Periodontology, Karnataka Lingayat Education Society (KLE) Veerendra Patil Khandalgi (VK) Institute of Dental Sciences, Belagavi, IND; 3 Periodontology, Maratha Mandal’s Nathajirao G. Halgekar Institute of Dental Sciences & Research Centre, Belagavi, IND

**Keywords:** dentinal tubules, scanning electron microscopy (sem), nano carbonate apatite (n-cap), diode laser, dentinal hypersensitivity (dh)

## Abstract

Background: Dentin hypersensitivity (DH) involves sensitive symptoms, because of exposure of the dentinal tubules. Various materials have been utilized to occlude dentinal tubules for the treatment of DH. Here is a comparative evaluation of nano-carbonate apatite (n-CAP), diode laser, and their combination over the occlusion of dentinal tubules.

Materials and method: Ten intact first premolars were used in this study, out of which 40 dentin disk specimens were obtained by hard tissue microtomy. Four study groups were formulated out of which one was the control group and the remaining three were test groups. Scanning electron microscopy (SEM) was done to evaluate the diameter of the dentinal tubules in each group.

Results: On examining data, it was observed that the mean diameter of dentinal tubules in four study groups of control, laser, n-CAP, and n-CAP + laser was found to be 3.40, 2.00, 0.46, and 0.02 respectively. This shows the significant reduction in the diameter of dentinal tubules in the test groups when compared with the control group.

Conclusion: Among all the measures used to see for a reduction in the diameter of dentinal tubules, the combination group was found to be most occluding, though each of the groups also had a significant reduction in the diameter of dentinal tubules. The present study showed that combination therapy offers a promising means of treating DH in a clinical setting when compared with the treatment of DH n-CAP containing dentifrice or laser irradiation alone.

## Introduction

Dentin hypersensitivity (DH) is one of the most common problems causing pain and discomfort following various thermo-mechanical, and evaporating stimuli at cevically exposed dentin [1]. Many studies have concluded that the prevalence of DH is as high as, one case among every seven individuals [2-5]. Conditions such as occlusal attrition, the recession of gingiva, abrasion, erosion following faulty oral hygiene practices, and treatments such as scaling and root planning (SRP), all can cause exposure of dentinal tubules [6]. In both situations of cervical erosion and attrition, the enamel is lost due to relevant habits of faulty brushing and traumatic bite and so on, which leads to exposure of underneath dentinal tubules and further hyperactive dentinal fluid movement to various bearable stimuli leading to aggravated sensitivity symptoms, which are common to both clinal scenarios. Other factors that can also seriously affect DH include age, sex, occlusal imbalance, oral pH being acidic, bacterial plaque accumulation, and the level of the patient’s oral hygiene practices [7]. It is evident that in non-sensitive dentin, almost all of the dentinal tubules are occluded, while sensitive dentin has a significant number of open tubules towards the surface [8].

According to Brannstorm's ‘‘hydrodynamic theory,’’ hydraulic changes in the tubular ﬂuid of exposed dentin are the main reason for direct stimulation to pulpal mechanoreceptors or indirect stimulation of odontoblasts [9]. This situation can also provoke some changes in the pulpo-dentinal complex which can further inﬂuence normal function of the peripheral nerves and all of this leads to inflammation of the pulp [10]. Major symptoms of DH include cognition of a short and sharp pain of sudden onset. While, in severe cases, episodes of high to low intensity, delayed, prolonged, and vague pain is experienced following stimulation [11].

Various materials have been employed to occlude the dentinal tubules for the treatment of DH. One of which, is hydroxyapatite (HAP). It is a biocompatible material that has been studied as a bone substitute in the field of medical science. Moreover, HAP combined with nanotechnology has new physical properties with reduced particle size [12]. Instead of that, nano-carbonate apatite (n-CAP) is more similar to the inorganic component of the teeth/bones, and has excellent biocompatibility, compared with the hard tissues [5,6]. Also, it was able to get well adsorbed on the exposed tooth and root surfaces because of their increased surface area and high surface energy. Recently, it has been reported that the dentifrice containing 20% of n-CAP, was the most effective in occlusion of the dentinal tubules when compared with other conventional desensitizing dentifrices.

Since the advent of ruby lasers in dentistry, many studies have used lasers for the treatment of DH. He-Ne, diode, CO2, Nd: YAG, Er, Cr: YSGG, and Er: YAG lasers are used as a potential modality for the treatment method of DH [13]. The mechanism of laser irradiation is known to occlude the dentinal tubules by partial melting and recrystallizing the dentin surface, which reduces the acid solubility of the surface and thus, provides long-term desensitizing effects [6]. Diode laser irradiation also leads to ultrastructural alterations in intra-radicular dentin ranging from modifying the smear layer by initial melting and partially occluding open dentinal tubules. n-CAP within the dentifrice can provide a biocompatible layer, which on laser irradiation when placed over open dentinal tubules, can improve the effectiveness of the treatment with minimal damage to the tooth surface, and in addition, the sensitivity will be reduced.

Hence, this study was conducted to estimate and compare the effectiveness of diode lasers, n-CAP dentifrice, and their combination over exposed dentinal tubules for their occlusion, which further leads to decreased dentinal hypersensitivity.

## Materials and methods

Source of data

Freshly extracted first premolars were collected from the Department of Oral and Maxillofacial Surgery at Maratha Mandal’s Nathajirao G. Halgekar Institute Dental College and Hospital, Belagavi, affiliated to Rajiv Gandhi University of Health Sciences, Bengaluru, India. The selection was based on inclusion and exclusion criteria.

Methods of data collection

Ten intact first premolars extracted for orthodontic treatment were used in this study. After extraction, the teeth were stored in a freezer at (-) 20°C as soon as possible until their use without any medium or solution.

Inclusion criteria

Fresh extracted first premolars for orthodontic treatment were to be selected for the study; teeth with no periodontal involvement would have no restorations or filling and fracture.

Exclusion criteria

Teeth that were decayed, teeth that were periodontally compromised, teeth in which roots were not completely formed, teeth having restorations or filling, and teeth that were fractured.

Sample preparation

After receiving approval (No. 1139) from the institutional ethical committee of Maratha Mandal’s Nathajirao G. Halgekar Institute of Dental Sciences & Research Centre, each tooth was sectioned horizontally below the cementoenamel junction with a diamond wheel disc for each tooth. The root portion of the tooth was first embedded with an acrylic resin into a wax mold. Each dentin specimen was wet grounded using silicon carbide paper (P600-1200) to expose the dentin surface. To minimize the differences within each group, four parts of 2 mm^3^ were cut from each specimen. This decreased the variation, as the control and the experimental groups will be selected from the same tooth. Forty dentin disc specimens were obtained from 10 teeth. Processed specimens were then etched to open the dentinal tubules. Specimens were allotted into control and experimental groups. Four groups were designed: control group (10 sections), n-CAP group (10 sections), laser-irradiated group (10 sections), and combination (laser + n-CAP) group (10 sections).

Procedure

The control group was left untreated after etching. The n-CAP group was manually tooth-brushed with n-CAP dentifrice. In the laser irradiated group diode laser of the wavelength of 810 nm, fiber diameter 0.04 mm, power 2 Watts, irradiation mode pulse mode, irradiation time 3-5s, pulse width 30 ms, interval 30 ms and noncontact mode (distance: 1-2 mm) was used. The maximum power density (Watts/cm^2^) delivered was up to 1.59 Watts/cm^2^ and the fluence energy of the beam was up to 10.61 joules/cm^2^. In the combination group tooth-brushing with the n-CAP dentifrice was followed by irradiation with 810 nm diode laser with the same specifications. The dentifrice used in this study contains 20% n-CAP (Daewoong Pharmaceuticals, Seoul, South Korea). Manual tooth brushing was performed with 50 back-and-forth strokes in linear motion for about one minute.

Sample evaluation

Specimen evaluation was done by scanning electron microscopy at the Indian Institute of Science (IISc), Bengaluru. Further, to quantify the degree of occlusion of dentinal tubules, specimens were evaluated using an image analyzer at 5000x magnification, in the range of 10 micrometers.

The statistical software program Statistical Package for Social Sciences (SPSS), version 21.0 (IBM Corp., Armonk, NY) was used for data analysis. The Kolmogorov-Smirnov test for normality was done to see if the study groups followed a normal distribution. Results were tabulated, and mean values and standard deviations were calculated. A comparison of four study groups with respect to the diameter of dentinal tubules was done by one-way analysis of variance (ANOVA). A pairwise comparison of the diameter of dentinal tubules in four study groups was done by Tukey's multiple posthoc test. A chi-square qualitative test was done to compare study groups with respect to codes of the status of the diameter of dentinal tubules.

## Results

**Figure 1:**
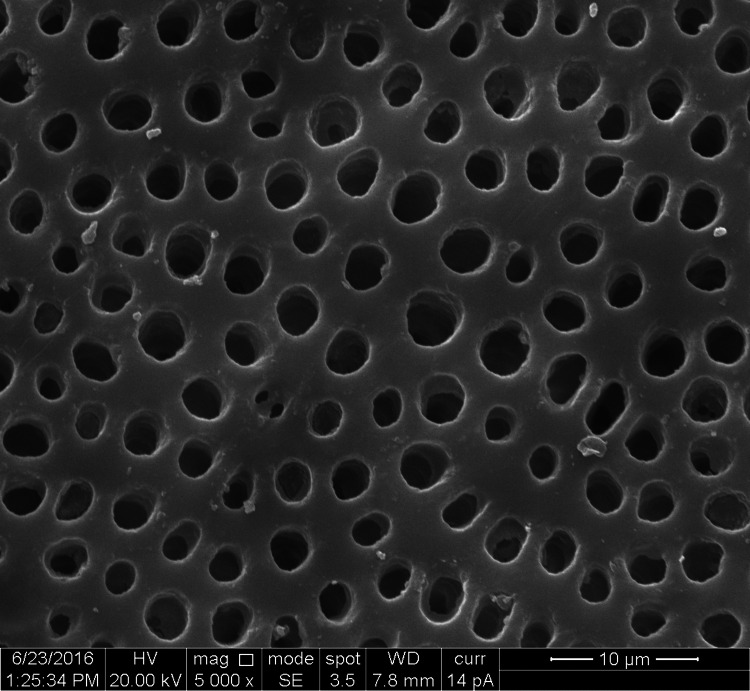
SEM image of the control group SEM: Scanning electron microscopy

**Figure 2:**
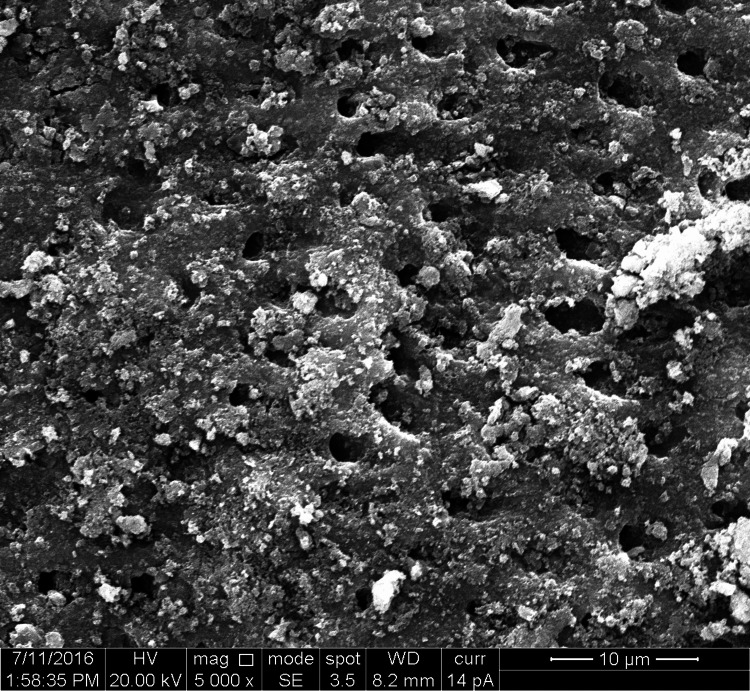
SEM image of n-CAP applied group n-CAP: Nano-carbonate apatite; SEM: Scanning electron microscopy

**Figure 3:**
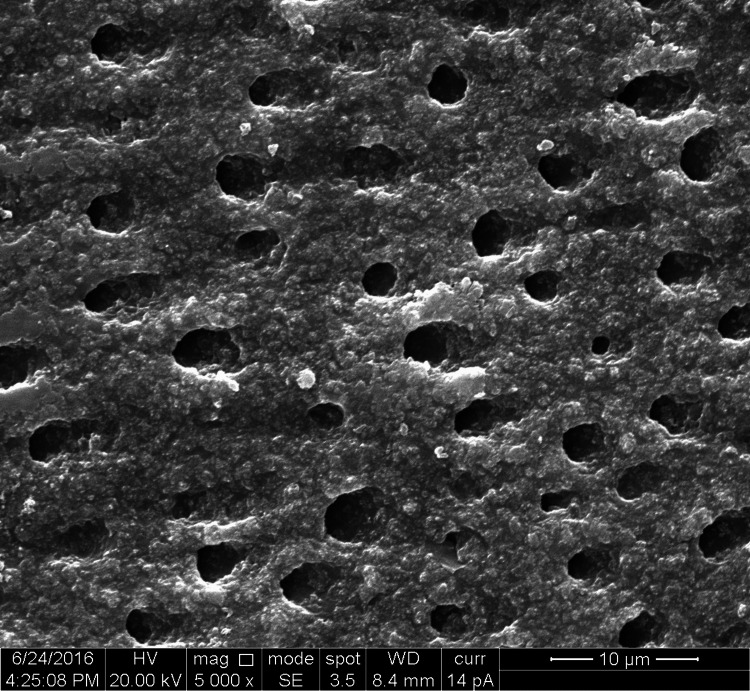
SEM image of the laser-irradiated group SEM: Scanning electron microscopy

**Figure 4:**
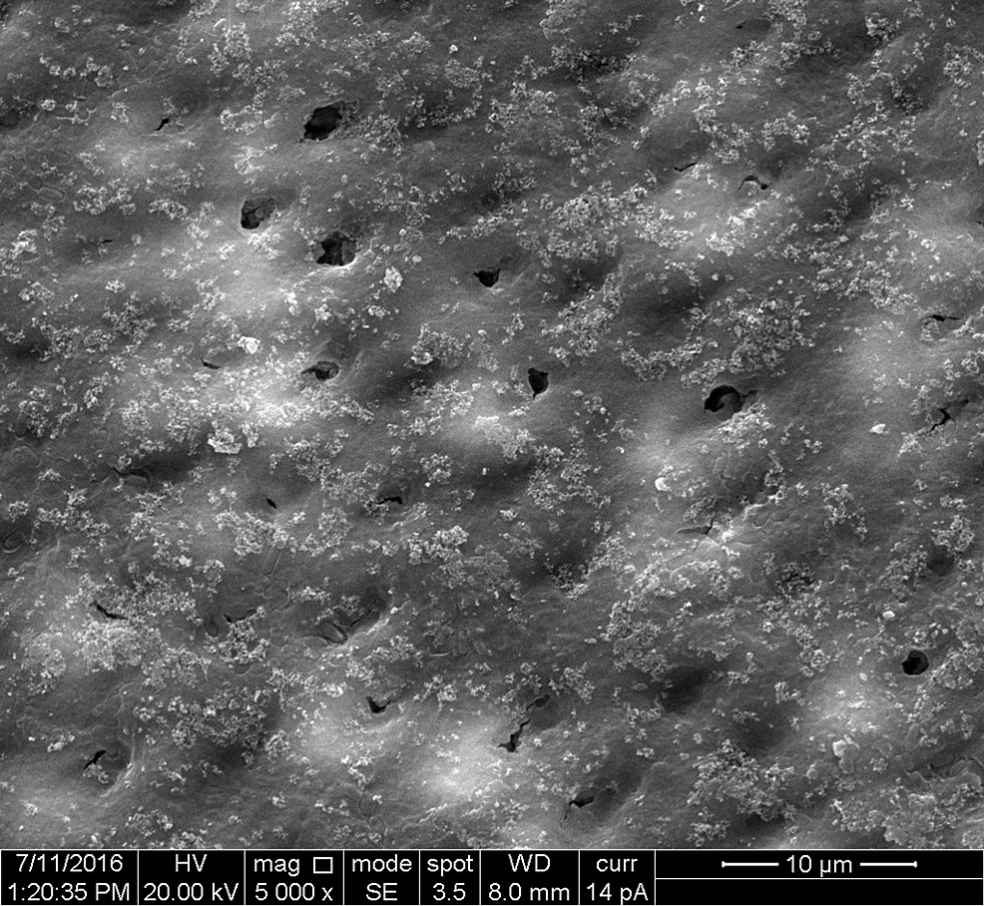
SEM image of combination n-CAP applied and laser-irradiated group n-CAP: Nano-carbonate apatite; SEM: Scanning electron microscopy

The study groups comprised 10 specimens of premolar teeth, four dentin disk sections prepared from each tooth, comprising a total of 40 dentin disc sections. Ten dentin disc specimens were distributed in each of the four groups. In each dentin disk section, the diameter of the five widest dentinal tubules was measured comprising 50 measurements in each of the groups and a total of 200 measurement values for the diameter of dentinal tubules (Figures 1-4).

The groups were organized as Group 1-control group (10 sections, 50 measurements of dentinal tubular diameter), Group 2-n-CAP group (10 sections, 50 measurements of dentinal tubular diameter), Group 3-laser-irradiated group (10 sections, 50 measurements of dentinal tubular diameter), and Group 4-combination (laser + n-CAP) group (10 sections, 50 measurements of dentinal tubular diameter). The codes which were used are shown in Table 1.

**Table 1:** For qualitative assessment of occlusion of dentinal tubules, their status of occlusion was provided three codes as not occluded, partially occluded, and up to completely or completely occluded

Codes	Status of occlusion of dentinal tubules
Code 1	Not occluded
Code 2	Partially occluded, 50% and more
Code 3	Upto complete or completely occluded, 90% to completely occluded

The diameter of dentinal tubules in the four study groups of control, laser, n-CAP, and n-CAP + laser have Kolmogorov-Smirnov coefficients (Z) as 0.7470, 0.5970, 0.5930, and 0.5830 respectively which follows a normal distribution. Therefore, the parametric tests were applied (Table 2).

**Table 2:** Diameter of dentinal tubules in four study groups n-CAP: Nano-carbonate apatite

Groups	Control	Laser	n-CAP	n-CAP + laser
Mean	3.4039	1.9978	0.4552	0.0226
SD	0.2229	0.1904	0.1352	0.0189
Absolute	0.2360	0.1890	0.1880	0.1850
Positive	0.2360	0.1890	0.1260	0.1850
Negative	-0.1410	-0.1460	-0.1880	-0.1150
Kolmogorov-Smirnov Z	0.7470	0.5970	0.5930	0.5830
P-value	0.6330	0.8680	0.8730	0.8850

Table 3 illustrates the degrees of freedom between the groups as 3 and within the groups as 36, comprising the total degree of freedom to be 39. The sum of squares between the groups was 71.43 and within the groups was 0.94, getting a total sum of squares to be 72.37.

**Table 3:** Degrees of freedom between the groups

Source of variation	Degrees of freedom	Sum of squares	Mean sum of squares	F-value	P-value
Between groups	3	71.43	23.81	911.14	0.0001
Within groups	36	0.94	0.02		
Total	39	72.37			

Dividing the sum of squares with respective degrees of freedom, the mean sum of squares was obtained, between the groups as 23.8114 and within the groups as 0.0261. The ratio of these two means of squares as obtained between the groups and within the groups provided the F-value, 911.1499, which rejects the null hypothesis, and the P-value was found to be 0.0001, which is significant (Table 4).

**Table 4:** Pairwise comparison for the mean ± SD of the diameter of dentinal tubules for control, laser, n-CAP, and n-CAP + laser groups n-CAP: Nano-carbonate apatite

Groups	Control	Laser	n-CAP	n-CAP + laser
Mean	3.40	1.99	0.45	0.02
SD	0.22	0.19	0.13	0.01
Control				
Laser	p=0.0001	-		
n-CAP	p=0.0001	p=0.0001	-	
n-CAP + laser	p=0.0001	p=0.0001	p=0.0001	-

Pairwise comparison of all the test groups with the control group resulted in a decrease in tubular diameter up to 1.9978 µm, 0.4552 µm, and 0.0226 µm for laser, n-CAP, and n-CAP + laser respectively, which is less than the control group. Similarly, pairwise comparison of n-CAP and n-CAP + laser group with laser group showed a decrease in tubular diameter up to 0.4552 µm, 0.0226 µm, which is less than laser group and pairwise comparison between n-CAP and n-CAP + laser groups showed a decrease in tubular diameter upto 0.0226 µm which is less than n-CAP group. In all of the above comparisons between the groups, p=0.0001 is significant (Table 5).

**Table 5:** Qualitative status of occlusion of dentinal tubules depicted as the % of dentinal tubules occluded in each of the groups n-CAP: Nano-carbonate apatite Chi-square = 101.5391, p = 0.0001

Codes	Control	%	Laser	%	n-CAP	%	n- CAP + laser	%	Total
Not occluded	10	100.00	0	0.00	0	0.00	0	0.00	10
Partially occluded	0	0.00	10	100.00	7	70.00	0	0.00	17
Upto complete or completely occluded	0	0.00	0	0.00	3	30.00	10	100.00	13
Total	10	100.0	10	100.00	10	100.0	10	100.0	40

Out of 10 dentin disk specimens in the control group, 100% of the dentinal tubules showed a nonoccluded state and were assigned code 1. Out of 10 dentin disk specimens in the laser group 100% of the dentinal tubules showed a partially occluded state and were assigned code 2. Out of 10 dentin disk specimens in the n-CAP group 70% of the dentinal tubules showed a partially occluded state and were assigned code 2 and 30% of the dentinal tubules showed up to complete or completely occluded state and were assigned code 3. All the groups thus comprised a total of 40 dentin disk specimens.

## Discussion

The dentinal tubules are typically 0.5 to 2 microns in diameter and contain plasma-like biological fluid that is connected to pulp. Depending on the depth, about 30,000 tubules can be found in a 1-millimeter square cross-section of dentin. It has been found that the number of open dentinal tubules per surface area in the exposed dentin surface of teeth with hypersensitivity can be eight times that of teeth non-responsive to stimuli [14,15].

The majority of studies report a tooth site predilection order for DH, highest at canines and first premolars, followed by incisors and second premolars, and, finally, molars, with the majority of sites being buccal and cervical. Both conditions have been shown to be more common on the left than on the right sides of the arches [16]. According to Grossman, treatment for DH must be fast, effective for long periods, easy to apply, non-irritant to the pulp, painless, stainless, and constantly effective [16]. Two prominent methods for the treatment of dentin hypersensitivity are tubular occlusion and blockage of nerve activity [17, 18]. Many materials and techniques have been used to treat DH, including specific dentifrices (containing agents like calcium, phosphate, potassium nitrate, and oxalates), adhesives, resin suspensions (glass ionomer cement), fluoride rinses and varnishes, periodontal membranes and laser irradiation [19]. Lasers have occluding effects on the dentinal tubules as well and their effects on nerve endings are also considered important. Although, occluding effects are still considered the most effective and definitive method for reducing tooth sensitivity. 

Considerable success has been achieved in reducing hypersensitivity, but still, most of the current modalities provide only temporary and unpredictable desensitization [20]. Therefore, new treatment modalities are needed, that have continued effectiveness for longer time periods, and also they should not have the aforementioned shortcomings and complications. An in vitro study was conducted to evaluate and compare the effect of n-CAP dentifrice, diode laser, and their combination over dentinal tubule occlusion to see if they are effective in reducing the diameter of dentinal tubules. Dentin disk specimens were allocated into four groups; control group (only treated by etchant to get open patent dentinal tubules), laser group (irradiated by 810 nm diode laser [21-23] post etching), n-CAP group (application of n-CAP dentifrice post etching) and combination of both n-CAP and laser group (application of n-CAP dentifrice post etching and subsequent application of 810 nm diode laser over the dentin disk specimen).

Desensitizing dentifrices include various active ingredients, which are effective in the occlusion of dentinal tubules. In our study, dentin disk specimens were prepared and etched with 37% phosphoric acid for 90 seconds. For the test groups, that is the n-CAP group and combination group of n-CAP and laser, n-CAP was applied after getting the dentinal tubules patent by application of etchant. After that 50 strokes of a soft bristle toothbrush were administered for 28 almost a minute on each test specimen. Carbonate apatite is a sort of calcium phosphate, closer to biological apatite that exists in dental and osseous tissues. It is widely used in the medical and dental fields and found to be highly biocompatible. In our study, dentifrice containing 20% n-CAP was used. Lee et al. reported that comparing the short-term use of desensitizing dentifrices in vitro, the dentifrice containing 20% n-CAP was the most effective in occluding the dentinal tubules [24].

Gholami et al. conducted a study for evaluating the occluding effects of Er, Cr: YSGG, Nd: YAG, CO2, and 810-nm diode lasers on dentinal tubules and found tubular diameter reduction in all laser groups, which was significant [25]. In our study for the laser test group, an 810 nm diode laser in non-contact mode with a power of 2 Watts was used to see its effect on the reduction in the diameter of dentinal tubules. Further, the results of dentinal tubules occlusion for the n-CAP group alone and the combination group of n-CAP and diode laser were compared over dentin disk specimens so prepared, keeping an etched, untreated dentin disk group as control.

In our study, we evaluated the mean diameters of dentinal tubules for each group. According to the results obtained, the mean diameters of dentinal tubules in the control group was 3.40 µm, which represents the tubular diameter of open and patent dentinal tubules. For the test groups of the diode laser, n-CAP, and the combination of both diode laser and n-CAP, the mean diameters of dentinal tubules were 2.00 µm, 0.46 µm, 0.02 µm respectively, which was less than the tubular diameter of the control group. The reduction in the mean diameter for the laser group showed that the 810-nm diode laser sealed tubules to a lesser degree when compared with other test groups but resulted in partial occlusion of dentinal tubules with respect to the control group with a reduction in the mean diameter of 1.40 µm. This will reduce the dentinal fluid movement and decrease hypersensitivity as mentioned by Rimondini et al. [26].

Comparing the reduction in the mean tubular diameter between the control and the n-CAP group, a reduction of 2.94 µm was found, leading to partial occlusion of dentinal tubules. However, the mean value shows a partial occlusion of dentinal tubules, while dentinal tubules in some of the dentin disk specimens showed almost complete occlusion after the application of n-CAP.

Comparing the reduction in the mean tubular diameter between the control and the combination of n-CAP and laser group, we found a reduction of 3.38 µm, which shows almost complete occlusion of dentinal tubules. This result is in accordance with the study conducted by Han et al, which shows that the combination group has the potential for enhancement of the dentinal tubule occlusion [27], but differs in the finding that the n-CAP group has more occluding effect than the combined group.

Obtaining the results from the in vitro study and comparing the quantifiable parameters of the results by one-way ANOVA test, we found that the combination group shows maximum occlusion of dentinal tubules followed by n-CAP group and diode laser group respectively. To find out the status of occlusion of dentinal tubules in terms of unoccluded, partially occluded, and up to complete or completely occluded, three codes were given as code 1, code 2, and code 3 respectively. In our study in terms of the percentage of occlusion in the tubular diameter of dentinal tubules, we found that in comparison with the control group of completely patent dentinal tubules, there was a reduction in tubular diameter by almost 60% in the laser group, more than 90% in n-CAP group and almost complete obliteration of dentinal tubules in combination of n-CAP and laser group.

Comparing the results in all the test groups with respect to the control group, we found the reduction in the mean diameter for laser, n-CAP, and combination of n-CAP and laser group to be 1.40 µm, 2.94 µm, and 3.38 µm respectively. Comparing, the reduction in the mean diameter between the test groups, it can be stated that the reduction in the tubular diameter caused by the application of n-CAP was 2 times than that of laser and the reduction in the tubular diameter caused by the application of both n-CAP and laser was almost 2.5 times than that of laser. The results so obtained in our study show significant variability in the diameter of dentinal tubules between test groups when compared with the control group as well as between each other. According to the results, it is evident that the combination of n-CAP and laser shows synergistic effects than the use of laser or n-CAP alone. Thus, the results of the study indicate occlusion and reduction in diameter of the open dentinal tubules either by applying laser, n-CAP as well as their combination therapy.

Limitations include that the penetration of desensitizing agents could have been evaluated by taking the longitudinal section of the dentin disk specimen. The brushing mechanism administered in our study was manual, which could have been made more reliable by using a V-8 cross-brushing machine for equal distribution of force. The present study showed that combined therapy (810 nm diode laser + 20% n-CAP containing dentifrice) can offer a promising means to treat DH in a clinical setting. Further, studies are needed in this context to test the effect of these treatment modalities over a period of time by providing an acid challenge for the sustainability of the treatment effect using an oral simulation model to simulate an in vivo situation and anticipate the same in clinical scenarios. Also, there are different imaging modalities for the measurement of dentinal tubules other than scanning electron microscopy, such as confocal microscopy and spectrophotometry, that can be employed and compared for further reliability and precision of the result. Making all of these limitations to our study and subject of further research and evaluation.

## Conclusions

In all of the test groups, there was an observable reduction in the diameter of dentinal tubules, All the employed measures as 810 nm diode laser, nano-carbonate apatite (n-CAP) containing dentifrice, and a combination of both laser and n-CAP are effective in reducing the diameter of open dentinal tubules. The combination group is most effective in occluding the dentinal tubules as almost all of the dentinal tubules were obliterated in that group. The n-CAP group showed more reduction in the diameter of the open dentinal tubules. 
